# Observations and Cases Illustrative of the Effects Produced by the Solar Heat upon the Human Body

**Published:** 1810-03

**Authors:** John Howship

**Affiliations:** Surgeon


### *Observations and Cases illustrative of the Effects produced by the Solar Heat upon the Human Body.*

By Mr. JOHN HOWSHIP, Surgeon.

THE numerous diseases to which the human frame is subjected, exposed as it is to the influence of the changing seasons in the various climates of the habitable globe, seem generally to depend either upon one or both of two causes; the first of which relates to the usual condition of the atmosphere as connected with high or low, moist or dry, confined or airy situations; the second arises from the direct influence of the heat of the sun, regulating the temperature of the air, and in a great measure, the excitement of the body. To the first of these may justly be referred the predisposition to, and even the production of many formidable diseases; but it is the second only, that will be the subject of consideration at present.

With regard to the effects of solar heat upon the human body, it may be observed, that those who in a temperate latitude, have usually enjoyed full health and strength, will upon removal to a warmer climate be very liable to suffer from any particular description of inflammatory attack to which there may have been constitutional predisposition; while, on the other hand, such as do not possess what is termed a good constitution, who never have been subject to inflammatory complaints, but have been considered to possess a weakly habit of body, do also generally experience very unpleasant consequences from a high temperature; many persons of the latter description complain, that their feelings are constantly those of irksome languor and depression; and from any continued exertion, or any accidental exposure to the sun, they will sometimes fall down suddenly, quite exhausted, and reduced to a state of temporary fainting and insensibility.

In the climate of the Mediterranean for instance, where Fahrenheit's thermometer, in the summer season, usually ranges between 70 and 86° in the shade, although not within the tropics, nothing is more common than such accidental complaints as arise simply from the heat of the sun operating variously upon various habits and in various constitutions; producing in some a violent paroxysm of highly inflammatory fever, or an attack of local inflammation; in others again, whose habit of body is weak and deficient, a sort of faintness, or a fit of sudden insensibility with languor, very generally preceded by some of the symptoms indicating an excessive debility; and it matters not whether the weakly condition of the habit is an original defect in constitution, or the accidental consequence of former disease of viscera.

Upon tedious garrison parades, in the Island of Minorca, it has very often happened that a young soldier, a recruit lately joined from England, has been overcome by the heat of the sun, and has fallen down in the ranks: afterwards complaining of a most violent heat and sharp pains shooting through his head, which state has been quickly followed by the heat of skin, dry tongue, wild delirium, &c. that attend inflammation of the brain, although he was in all respects well when he first came upon the parade.

In precisely the same manner it has very frequently happened, that the most healthy men have been overpowered by the force of the meridian heat of the sun. It has been necessary to carry them off from the parade; and upon inquiry, it has been found, that they have either had a sudden attack of the most insupportable pain, prickling and darting through every part of the chest, with very great oppression, so as almost to prevent their breathing at all, or they have complained of a severe and fixed pain in the side, somewhere under the margins of the ribs, or near the ensiform cartilage, catching their breath in inspiration, and connected with an acute pain at the shoulder.

The force with which the sun is capable of acting upon the human body, is truly astonishing; it is such, as will very readily explain its occasionally exciting inflammatory action, either local or general, in the system, even allowing, that in some instances, predisposition is not taken into the account.

The effect of heat upon weakly persons is that of a stimulus, which, without exciting any excessive action in particular organs, may operate merely as a power capable of producing a sudden exhaustion of irritability in the body. It seems to be evidently upon this principle, that a soldier has, in many instances, suddenly fallen down in the ranks, after having complained of a swimming in his head, followed by a general state of excessive and inexpressible failure of strength over every part of his body; or upon being carried from the parade, he has been found pale, languid, and nearly senseless; every voluntary muscle, both of body and limbs, being involved in a state of universal tremor, or faint vibration, which has gradually subsided, in proportion to the readiness with which the system has recovered its due tone, and the man has thus been restored.

But

But when the previous state of constitutional weakness has been very excessive, and the force of the solar rays also exceedingly great, the application of this powerful stimulus has very repeatedly been productive of a total and absolute exhaustion of the irritable or vital principle. The poor man has been struck down, almost as if by lightening; upon being carried into the shade, every possible exertion has been used to restore him to life, but in vain, and the man has died. It is however proper to observe, that such serious events as these are not of frequent occurrence, and are incident almost exclusively to such persons as have been long known to lead a life of intemperance, in whom consequently a state of great indirect debility must habitually have prevailed.

One of the most frequent immediate effects of the solar heat acting in force upon the living body, is the production of an inflammation; the stimuli acting either upon the external surface of the body, or else through the surface upon some of the contained viscera, any of which are liable to be thrown into a state of high excitement from the power of this most active agent.

Thus, in some cases, the heat of the sun has produced a temporary redness upon some portion of the surface of the body, raising the cuticle in vesications; while in other instances, troublesome inflammatory actions have been the consequence. In the year 1804, one of the soldiers of the 13th regiment of foot, then in garrison at Gibraltar, while engaged in washing his shirt upon the rocks at the seaside, was not at the time aware of the day being particularly hot, but his back and shoulders having been exposed to the action of the sun, before the evening a very violent and alarming degree of inflammation took place upon the integuments, attended with severe shooting pains, darting through the spine forward to the sternum, producing great distress in breathing. In this case, by the adoption of prompt measures, the inflammation was prevented from doing any serious mischief in the breast, but in spite of every exertion, a slough formed upon the back, of considerable extent, aud there was for some time a doubt whether the man would recover.

Independant however of those immediate effects enumerated above, the heated atmosphere of a warm climate may be considered to operate, though silently and slowly, yet rarely towards the production of any great changes in the state of constitution, in every individual who has formerly been resident in a more temperate climate.

The peculiar diathesis to the establishment of which a residence in a warm climate seems favourable, is a certain modification of irritability, manifested in the evident tendency to some particular forms of nervous disease, but not to others. That such a diathesis may exist, dependant upon temperature alone, is rendered extremely probable by the fact of its being present in the warm, but not in the more temperate seasons, in the same climate. Thus, in the Mediterranean, in the month of October 1805, after the late Lord Nelson's glorious victory at sea, near 400 wounded sailors and marines were landed at Gibraltar, out of which number, as many as eighteen died of lock jaw and tetanus; while it was clearly ascertained, that in the month of December 1803, after the Toulon expedition, more than 400 wounded men were sent on shore for treatment in the naval hospital of that garrison, and from this larger number of surgical cases, there was only one solitary example of tetanus.

### *A Case of Coup de Soleil.*

M. Kinnear, private in the 17th regiment of light dragoons, a strong and healthy man, twenty-two years of age, was on board one of the transports carrying his regiment out to South America, in the year 1806. From a deficiency in the supply of water, and the want of refreshments for the troops, it was judged expedient that the fleet should put in at St. Jago, off Cape de Verd. That island is situated in north latitude, and the fleet came to an anchor in the month of December; consequently, they arrived in the coolest season of that climate.

The heat of the sun was nevertheless very great, the thermometer generally being in the day-time as high as 80°; and, in the shade, and throughout the night, never below 78°.

On December the 14th, this man had been lying upon the deck after dinner, in conversation with several others, about two in the afternoon; about this time he felt himself drowsy, and soon after fell asleep. He lay exposed to the heat of the sun for about an hour, and then awoke, with excruciating pains in all his bones, together with a most violent shivering fit, so that he could scarcely stand up; added to these complaints, he had an intolerable degree of pain in his head, which almost took away his senses. He said that his head was "bursting" with horrible pain, darting and throbbing through his brain. The eyes were heavy heavy, and the conjunctive membrane of a dirty red colour; the countenance in general full, and much flushed.

About his chest he suffered great distress; the most violent stitches of pain shooting through "every part" of the breast. The pains that were in his arms and legs, also, were very acute and excessively severe.

In this state he made a shift to creep down between decks, and got into his birth; here the violence of the trembling gradually subsided, and a little time after he got some repose. He slept till about six o'clock in the evening, when he was accidentally disturbed, and found himself still very hot. But it is remarkable, that this heat was unattended with thirst, or the least sensation of heat in the mouth. He was, at six o'clock in the evening, reported sick by the serjeant of his troop, and had still most of the above symptoms upon him; dreadful pain in the head and great heat of skin, and universal pain everywhere.

The heat of skin however was now, in some degree subsiding into a state of perspirable moisture. An emetic of ipecacuanha, so directed as that it should operate very mildly, was ordered for him; this answered very well, and before midnight his stomach was cleared, and the bowels, as well as the surface, sufficiently relaxed. The pain in his head continued through the night so distractingly severe, that he was at times nearly delirious; and when his bowels were disturbed by the operation of the medicine he had taken, it was only by the assistance of two men that he could be removed, as he appeared neither to have power nor reason to enable him to direct his steps.

Early the following morning he was seen, and was found to be greatly relieved; the stitches through the breast being much less frequent, and much diminished in severity. The pain in his head, also, was very much alleviated. The pulse had been in the first instance much quickened, although not particularly hard; but it was, by this time, perfectly restored to its former state of tranquillity, beating only ninety in the minute.

The pain in the head from this time continued to diminish, but he was in point of strength more reduced than can be believed; and was for several days afterwards confined to his birth from mere weakness, being unable to support himself upon his feet. This man recovered.

### *A Case of Coup de Soleil.*

J. Gready, a strong healthy lad, nineteen years of age, a private in the 17th light dragoons, upon the 22d of December, 1806, was on shore at Porta Praya, St. Jago, with the fatigue party upon the sands, for the purpose of assisting in watering the fleet. He was employed in rolling the empty casks on shore from the boats, and in rolling them back again through the shoal water to the boats again, after they were filled. At this duty he was nearly naked, having on only a pair of loose canvas trowsers; and he was of necessity constantly in the salt water. With the party he returned on board at the usual hour in the evening, and was almost immediately afterwards seized with a violent trembling in his limbs and body; attended with so severe a pain in the head, that he was every now and then delirious. According to his own mode of expression, his head throbbed as if it was in the act of splitting. Consistent with this persuasion, he requested that his head might be bound round as tight as possible with a handkerchief. He also suffered extremely from pains darting violently through every part of his breast, and about the sternum especially. With these complaints his pulse was quickened, but was perfectly soft and small: the tongue was rather disposed to whiteness, and he complained of being very thirsty. An emetic of the ipecacuan powder was ordered for him; which operated so powerfully in his favour, that by the following morning he was almost completely relieved from the pains, with which he had been afflicted in his head and in his chest.

Upon the second day afterwards, the only remains of his complaints were about the breast. He had still a stitch of sharp pain now and then, and a troublesome cough, which was not perfectly removed for several weeks after the accident; but eventually he recovered.

### *A Case, in which fatal Disease arose from a Coup de Soleil.*

A soldier in the garrison of Gibraltar, while on a working party, weary and fatigued, laid down upon his face, and soon fell asleep, at a time when the day was clear, and the sun hot. He soon, however, awoke in great pain, and was confined to his bed in the hospital for six weeks after, with no other complaint than a constant and distracting pain in and about his forehead. At the completion tion of this period, he said that he found himself getting better; in short, being a good soldier, he volunteered going to his duty, in preference to being considered any longer an invalid. Nothing more was heard of him by the surgeon for six months; when the pain in his head became again so urgent, that he was at last obliged to give way to it, and report himself. He was a second time admitted into the hospital, and the pain became for a considerable length of time remittent; returning with aggravated severity every morning, and sometimes missing a morning, or changing its hour of increased violence. Still, however, the constitutional health continued unimpaired; the appetite had been constantly good, and the pulse undisturbed; the skin always cool and perspirable, and the tongue moist.

About two months after his second admission into the hospital, he felt himself growing worse in the night; he became slightly delirious, and soon after this expired.

Upon examination of the head after death, it appeared that an universal increase of vascularity had taken place within the cranium. In no part of the cerebrum, however, could any decided disease be discovered. Upon raising the tentorium, however, the surface of the cerebellum was found to be in a state of disease; an ulcerated superficial cavity being found, that was about the size of a shilling, containing a thin ichorous matter. The pia mater and vascular tissue were destroyed and lost on this part, and the corresponding surface of the dura mater discoloured.

*Mill-street, Hanover-square,*

*January* 16, 1810.

